# Factors related to dropout in integrative oncology clinical trials: interim analysis of an ongoing comparative effectiveness trial of mindfulness-based cancer recovery and Tai chi/Qigong for cancer health (The MATCH study)

**DOI:** 10.1186/s13104-020-05172-5

**Published:** 2020-07-17

**Authors:** Devesh Oberoi, Katherine-Ann L. Piedalue, Hassan Pirbhai, Steven Guirguis, Daniel Santa Mina, Linda E. Carlson

**Affiliations:** 1 Psychosocial Resources, Tom Baker Cancer Centre, Holy Cross Site Phase I, 2202-2 St SW, Calgary, AB T2S 3C1 Canada; 2 Cancer Rehabilitation and Survivorship Program, Princess Margaret Cancer Centre, B PMB 130, 200 Elizabeth St, Toronto, ON M5G 2C4 Canada; 3 Faculty of Kinesiology and Physical Education, University of Toronto, 55 Harbord Street, Toronto, ON M5S 2W6 Canada; 4 Psychosocial Oncology, Department of Oncology, Cumming School of Medicine, University of Calgary, Health Sciences Centre Foothills Campus, 3330 Hospital Drive NW, Calgary, T2N 4N1 AB Canada

**Keywords:** Adherence, Loss to follow up, Waitlist control, Clinical trial, Behavioural trial, Mindfulness, Taichi/Qigong

## Abstract

**Objective:**

To examine the factors associated with loss to follow-up (LTFU) in an ongoing preference-based randomized waitlist controlled trial of mindfulness-based cancer recovery (MBCR) and Taichi/Qigong (TCQ) for cancer survivors (the MATCH Study). Hierarchical logistic regression was used to determine the factors associated with LTFU. Predictors included adherence to treatment, preference vs. randomized, type of intervention (MBCR vs. TCQ) and program timing (immediate {IM} vs. waitlist control {WLC} group).

**Results:**

Data indicated that randomization to the WLC group and, once in the intervention, low adherence were the main predictors of LTFU. Participants in the WLC group were 4 times more likely to be LTFU post-randomization [OR 3.96, 95% CI 2.08–7.56, p < 0.005] than those in the IM group. Participants showing low adherence to treatment were 6 times more likely for LTFU post-intervention [5.87 (2.57–13.400; p < 0.005] and 4 times more likely for LTFU 6 months post-intervention [OR 3.93, 95% CI 1.53–10.02, p = 0.01].

## Introduction

To accurately evaluate the efficacy of new treatments in randomized controlled trials (RCTs) and to ensure the generalizability of the results, participants should have high adherence and a minimal loss to follow-up (LTFU) [1–3]. There is a growing body of literature highlighting high rates of LTFU and non-adherence in behavioural interventions [4]. Non-adherence is the extent to which the participants fail to follow the treatment recommendations [5], while LTFU is failure to complete study assessments. Although both adherence and LTFU are grounded in participants’ intrinsic motivation to fulfil study requirements [6, 7], it is unclear how the two are related. Given the paucity of data, the goal of the current analysis was to examine how participants’ adherence, study design elements and individual factors predicted LTFU in our ongoing trial, the MATCH study (Mindfulness and Taichi/Qigong in cancer health) (ClinicalTrials.gov: NCT02801123) [8].

The MATCH study is a multi-site (Calgary, AB, and Toronto, ON) preference-based randomized waitlist controlled (WLC) comparative effectiveness trial of two mind–body intervention (mindfulness-based cancer recovery (MBCR) and taichi/qigong (TCQ) in cancer survivors [8]. Participants chose their preferred intervention or to be randomized. The WLC group acted as the control for the immediate (IM) group and received the intervention after 3–4 months. Patients in the preference arms got their preferred intervention, and were randomized 2:1 to IM or WLC. Patients in the randomized arm were randomized 1:1 to either intervention, then 2:1 to IM or WLC. The 2:1 ratio was used for the waitlist to enhance recruitment, since we suspected waiting may negatively affect recruitment and dropout rates. This would allow for a large enough sample size for the control group to conduct primary data analyses and more people in the intervention groups to improve power for planned subgroup and mediation analyses.

We sought to examine the relative contribution of: (1) program adherence and study design-related characteristics; (2) assignment to the WLC vs. IM group; (3) preference vs. being randomized, on LTFU. The current study is an interim analysis of data from the ongoing MATCH study. The findings may inform possible adaptations to the study design as well as help optimize the design of future RCTs involving psychosocial interventions to minimize LTFU.

### Hypotheses

1. Participants with low adherence to the intervention will have higher LTFU than those with high adherence
2. Participants in the WLC group will have higher LTFU than those in the IM group
3. Participants in the preference arms will have lower LTFU than those in the randomized arms

## Main text

### Methods

Participants were assessed in-person at pre-intervention (IM and WLC), post-intervention or post-wait (IM and WLC), post-intervention once the WLC got the program (WLC only), and 6-months post-intervention (IM and WLC). Participants attended weekly group sessions in-person and were instructed to do 30–45 min of home practice daily and record weekly practice. The study utilized weekly attendance and home-practice logs (HPL) to track adherence. The primary outcome of the study is Total Mood Disturbance post-intervention. Other measures recorded at each assessment included quality of life, psychological functioning, cancer-related symptoms, and physical functioning. Participants were also required to collect saliva samples at home and report to a blood laboratory to provide blood samples at each assessment. A detailed description of the trial methodology has been published elsewhere [8].

### Methodology

#### LTFU

Participants were considered LTFU if they did not provide data at: (1) post-randomization (participants were randomized, but did not attend the program); (2) post-intervention, or; (3) 6-month post-intervention follow-up.

#### Adherence

Adherence to intervention was tracked using class attendance (CA) and HPL completion. Low-attendance and low home-practice was defined as < 50% attendance and HPL completion. The 50% cut-off was somewhat arbitrary, but consistent with our previous definition of “program completers” as those who attended at least half of the sessions [9]. We have also seen in previous research that by the halfway point most participants have responded to the intervention [10]. A single variable called “adherence” was created by combining class attendance and HPL and had four levels:

1. Low attendance, low HPL
2. Low attendance, high HPL
3. High attendance, low HPL
4. High attendance, high HPL

During the intervention, class attendance and HPL was recorded weekly by the facilitator and participants, respectively.

### Participants

All MATCH study participants (n = 274) whose 6 months follow up assessment were due before the start of this interim data analysis (October 2019) were included.

### Statistical data analysis

Descriptive statistics included rates of adherence and LTFU at all follow-up assessment times, participants’ socio-demographic characteristics and their baseline psychological distress levels measured by the Distress Thermometer [11].

#### Factors associated with LTFU

We conducted hierarchical regression analysis with LTFU as the dependent variable. Models were created for (i) post-randomization, (ii) post-intervention and (iii) 6-month follow up.

Independent variables included: (i) adherence (ii) program start timing (IM vs WLC) (iii) preference (preference vs randomized) and (iv) program type (MBCR vs. TCQ).

Covariates: Participant characteristics (age, sex, education, employment, marital status, baseline distress).

Bivariate analyses examined the associations between each of the independent variables and the dependent variables. Based on the purposeful selection of variables for model building [12], only those factors significantly associated with the outcome variable (p ≤ 0.2) were entered into multiple logistic regression models to assess their relative contributions. Additionally, variables that were considered clinically relevant (baseline distress) were also entered. Odds ratios (OR) and 95% confidence intervals (CI) and associated *p* values were reported. The multivariate models controlled for covariates.

#### LTFU post-randomization (Model 1)

A three-step model was used. First, we entered the type of intervention (MBCR/TCQ), then participant characteristics and lastly study-design related factors into the model. The final model accounted for the relative association of all independent variables entered in the model with LTFU.

#### LTFU post-intervention (Model 2) and at 6-month follow-up (Model 3)

A 4-step hierarchical logistic regression analysis examined the relative role of participants, study-design related characteristics and participant adherence with the outcomes. First, we entered the program type (MBCR/TCQ). In steps 2 and 3, we entered participant and study-design related variables respectively. Lastly, adherence was entered to investigate its impact over and above the study-design related characteristics.

#### Sample size

As per Green’s estimate [13] for up to 10 independent variables in logistic regression, and to achieve a medium effect size and a power of 0.80 (α = 0.05), a sample of 117 was required. Statistical analyses were performed using IBM SPSS (version 25).

### Results

#### Participants

Over 75% of the participants were females (n = 209), and nearly three quarters (n = 197) were 46–75 years of age. Participants were well-educated, with 85% (n = 236) having over 13 years of formal education. Over 50% were employed (full or part-time). Nearly 3/4 of participants (n = 208) had self-reported distress levels ranging from 4 to 6 (out of 10). Participant program timing closely reflected the 2:1 allocation for IM (n = 179) and WLC (n = 94), over 3/4th of participants (n = 208) indicated a preference for intervention, and participants who had no preference were randomized (n = 64) equally into either intervention. The participant characteristics are reported in Table 1. Participants age, sex, education, marital status, employment, and distress (all p > 0.05, data not shown) were similar in the IM vs. WLC and preference vs randomized groups.

**Table 1:** Participant and study characteristics

Variable	Frequency (%)
Age (years) [Mean (SD)] 60.50 (11.57)	
< 45	57 (21)
46–60	103 (38)
61–75	94 (34)
> 76	19 (7)
Sex	
Females	209 (77)
Years of education [Mean (SD)] 15.64 (4.32)	
Employment	
Unemployed/Disability	26 (12.5)
Retired	71 (37.0)
Employed (part or Full time)	105 (50.5)
Distress scores (range 4–10) (mean 5.39 ± [1.38]	
4	97 (35.5)
5–6	111 (40.5)
> 7	26 (24)
Marital status	
Single/divorced/widowed	66 (31.7)
Married/Co-habitation	142 (68.3)
Group allocation	
MBCR	125 (46.8)
TCQ	142 (53.2)
Preference allocation	
Preference	208 (76.5)
Randomized	64 (23.5)
Cohort allocation	
Immediate	179 (65.6)
Delayed	94 (34.4)

#### Adherence and LTFU

The mean class attendance and completion of HPL of all participants was 71% and 54.5%, respectively. About 17% of participants (n = 47) had low attendance and low HPL, 9% (n = 24) low attendance and high HPL, 35% (n = 96) high attendance and low HPL and 39% (n = 107) high attendance and high HPL. Nearly a fifth of our participants (19%, n = 52) were LTFU post-randomization. Of those who participated in the program, 43% were LTFU at post-intervention (n = 96) and 46% (n = 102) at 6 months follow up.

### Factors associated with LTFU in different conditions in bivariate and multivariate models

The results of the bivariate analyses between each of the independent variables and the three dependent variables are reported in Table 2. The table reports the association of the WLC with LTFU at all time points, and the association of adherence with LTFU at post-intervention and 6 months follow up.

**Table 2:** Results of univariate regression for loss to follow up

Variable	LTFU post-randomization	LTFU post-Intervention	LTFU 6 month FU
Age			
< 45	1	1	1
46–60	1.22 (0.51–2.91); p = 0.65	1.38 (0.71–2.68); p = 0.34	1.54 (0.73–3.22); p = 0.26
61–75	1.23 (0.51–2.97); p = 0.65	1.27 (0.65–2.49); p = 0.49	1.05 (0.49–2.25); p = 0.89
> 75	2.46 (0.74–8.18); p = 0.14	1.54 (0.54– 4.41); p = 0.42	1.68 (0.51–5.51); p = 0.39
Sex			
Males (ref)	1	1	1
Females	1.32 (0.62–2.81); p = 0.48	1.31 (0.73–2.32); p = 0.36	1.09 (0.59–2.04); p = 0.76
Education	0.95 (0.88–1.02); p = 0.17	0.97 (0.92–1.03); p = 0.33	0.94 (0.87–1.01); p = 0.08
Marital status			
Married/cohabiting	1	1	1
Single/divorced/widowed	0.86 (0.37–1.98); p = 0.73	1.08 (0.58–2.00); p = 0.80	1.42 (0.74–2.73); p = 0.29
Employment status			
Unemployed/disabled	1	1	1
Retired	0.73 (0.20–2.59); p = 0.63	1.42 (0.51–3.99); p = 0.51	2.60 (0.79–8.56); p = 0.12
Employed (PT/FT)	1.14 (0.35–3.700; p = 0.83	2.14 (0.79–5.76); p = 0.13	3.94 (1.24–12.55); p = 0.02
Baseline distress	1.04 (0.84–1.29); p = 0.72	1.07 (0.89–1.270; p = 0.45	1.06 (0.87–1.28)
Program type			
MBCR	1.39 (0.74–2.58); p = 0.30	1.32 (0.81–2.16); p = 0.26	1.12 (0.66–1.90); p = 0.68
TCQ (ref)	1	1	1
Program preference			
Randomized	1.18 (0.58–2.39); p = 0.65	1.08 (0.62–1.90); p = 0.78	.64 (0.34–1.21); p = 0.17
Preference (ref)	1	1	1
Program timing			
IM (ref)	1	1	1
WL	4.08 (2.16–7.71); *p < 0.005*	2.23 (1.34–3.70); *p = 0.002*	2.56 (1.44–4.54); *p = 0.001*
Adherence	N/A		
LL (CA = low, HPL = low)		8.29 (4.12–16.66); *p < 0.005*	5.84 (2.71–12.59); *p < 0.005*
LH (CA = low, HPL = high)		6.15(2.15–17.63); *p = 0.001*	3.94 (1.23–12.59); *p = 0.02*
HL (CA = high, HPL = low)		1.12 (0.57–2.47); p = 0.64	1.82 (0.89–3.63); p = 0.09
HH (CA = high, HPL = high) (ref)		1	1

### Multivariate associations

#### LTFU Post-randomization (Model 1)

Program starting time (IM vs. WLC group) was a significant predictor of LTFU post-randomization, with significantly higher odds of LTFU in the WLC group participants [OR 3.96 (2.07–7.55), p < 0.005] (Table 3). Table 3 reports the association of WLC with LTFU post randomization and post-intervention.

**Table 3:** Results of multiple regression for loss to follow up

Steps	LTFU post-randomization	LTFU post-intervention	LTFU 6 month FU
Program type			
MBCR	1.39 (0.74–2.58); p = 0.30	1.04 (0.58–1.88); p = 0.89	1.04 (0.55–1.95); p = 0.91
TCQ (ref)	1	1	1
	R^2^ 0.007	R^2^ 0.000	R^2^ 0.000
Program type			
MBCR		1.10 (0.60–2.03); p = 0.74	1.13 (0.59–2.18), p = 0.71
TCQ (ref)		1	1
	N/A^a^		
Employment			
Unemployed/disabled		1	1
Retired		1.58 (0.55–4.49); p = 0.39	2.92 (0.86–9.92); p = 0.09
Employed (PT/FT)		2.22 (0.82–6.04); p = 0.12	4.30 (1.32–13.98); p = 0.02
Education			0.95 (0.88–1.02); p = 0.15
		R^2^ 0.021	R^2^ 0.074
Program type			
MBCR	1.40 (0.73–2.67); p = 0.31	1.11 (0.60–2.03); p = 0.74	1.10 (0.57–2.13); p = 0.77
TCQ (ref)	1	1	1
Employment		1	1
Employed (PT/FT)		1.47 (0.51–4.26); p = 0.47	2.84 (0.83–9.68); p = 0.09
Unemployed/disabled		2.14 (0.78–5.91); p = 0.14	4.21 (1.29–13.73); p = 0.02
Retired			0.94 (0.88–1.01); p = 0.95
Education			1
			1.57 (0.77–3.19); p = 0.26
Program timing	1	1	1
IM (ref)	3.96 (2.08–7.56); *p < 0.005*	2.23 (1.19–4.14); *p = 0.012*	0.48 (0.20–1.14); p = 0.09
WL			
	R^2^ 0.112	R^2^ 0.065	R^2^ 0.106
Program type			
MBCR		1.11 (0.56–2.16); p = 0.77	1.24 (0.62–2.51)
TCQ (ref)		1	1
Employment			
Employed (PT/FT)		1	1
Unemployed/disabled		2.15 (0.67–6.93); p = 0.19	(3.65.97–13.73); p = 0.05
Retired		3.21 (1.05–9.81); *p = 0.04*	5.33 (1.47–19.29); p = 0.01
Education			0.95 (0.88–1.03); p = 0.19
Program timing			
IM (ref)		1	1
WL		1.37 (0.67–2.78); p = 0.39	1.02 (0.46–2.28); p = 0.95
Adherence			
LL (CA = low, HPL = low)		5.87 (2.57–13.40); *p < 0.005*	3.93 (1.53–10.02); *p = 0.04*
LH (CA = low, HPL = high)		8.15 (2.41–27.58); *p = 0.001*	5.02 (1.26–19.96);* p = 0.02*
HL (CA = high, HPL = low)		0.99 (0.39–2.52); p = 0.99	1.37 (0.57–3.27); p = 0.48
HH (CA = high, HPL = high) (ref)		1	1
		R^2^ 0.251	R^2^ 0.192

The program start timing (i.e. whether the participants were in IM or WLC) was associated with LTFU at all the three time points

^a^N/A: Not applicable

#### LFTU Post-intervention (Model 2)

Adherence was the only significant predictor of LTFU at post-intervention accounting for 86% (15.7%/18.2%) of the total variance explained by the model (R^2^ total = 0.18). With reference to those with high CA and high HPL, those with low CA and low HPL [OR 4.67, 95% CI 1.75–12.46, p = 0.002], and those with low CA and high HPL [OR 7.75, 95% CI 2.22–27.06, p = 0.001] had higher odds of LTFU at post-intervention. Those with high CA and low HPL [OR 0.97, 95% CI 0.38–2.46, p = 0.96] were similar to those with high CA and high HPL indicating that low CA was a more significant indicator of LTFU than HPL. Program timing was non-significant (p > 0.05) (Table 3).

#### LFTU 6-month follow-up (Model 3)

Adherence was significantly associated with LTFU at 6 months follow-up, accounting for 48% (8.6%/18%) of the total variance explained by the model (R^2^ total = 0.18). Compared to those with a high CA and high HPL, those with low CA and low HPL [OR 4.14, 95% CI 1.62–10.52, p = 0.003], and those with low CA and high HPL [OR 5.60, 95% CI 1.43–21.84, p = 0.01] had higher odds of LTFU at PI. Those with high CA and low HPL [OR 1.54, 95% CI 0.66–3.58, p = 0.32] were similar to those with high CA and high HPL indicating that class attendance was a more important indicator of LTFU than HPL completion. Program timing was non-significant (p > 0.05) (Table 3).

## Discussion

Randomization to the WLC group was the main factor influencing dropout immediately after randomization; if participants stayed after randomized to the waitlist or immediate groups, then the next predictor of LTFU was adherence to intervention, specifically class attendance. Preference for intervention or lack thereof was not associated with LTFU. WLC participants had four times higher odds of LTFU post-randomization compared to those in the immediate group. This finding confirmed our hypothesis and was consistent with past research reporting a higher rate of LTFU in WLC groups [14, 15]. Evidence suggest that participants are motivated to start immediately, but motivation may diminish over time [16].

For LTFU post-intervention and at 6-month follow-up, the start time (IM vs. WL groups) was not a significant predictor. At these time points, low adherence was associated with significantly higher odds of LTFU. Participants who had low-CA regardless of HPL completion were significantly more likely to be LTFU compared to those with high-CA and high HPL. It is plausible that if participants attend more classes, they may have higher levels of social connectedness, and perceived benefits from the program and therefore continue attending [17], thereby reducing LTFU incidents. They also may be more conscientious or in better health overall, leading to both better adherence and completion of study requirements. Findings are supportive of the potential role of improved adherence, particularly class attendance, in reducing LTFU post-intervention and in follow-up assessments.

The preference for intervention (preference vs. randomized groups) was not associated with LTFU at any of the three-time points evaluated. It’s possible that in contrast to traditional RCT designs, participants in the randomized arm were not forced into any of the study arms against their true preference. High risk of LTFU in the WLC group is a sign that researchers should reconsider the inclusion of the WLC group in the study unless it is methodologically necessary. In a WLC trial, only participants who strongly agree to the possibility of being randomized to the WLC group should be enrolled.

## Limitations

As it was an ongoing trial, we could not comment on the effectiveness of the intervention and its impact on LTFU. Our sample was relatively homogenous as the proportion of female participants was substantially higher. We did not explore the role of cost and logistical challenges involved in attending these assessments, which include a comprehensive battery of psychological, physical, and physiological cognitive, and laboratory tests in the LTFU.

## Data Availability

The datasets used and/or analyzed during the current study are available from the corresponding author on reasonable request.

## Abbreviations

CA: Class attendance
HPL: Home practice logs
IM: Immediate
LTFU: Loss to follow-up
MATCH: Mindfulness and Taichi/Qigong in cancer health
MBCR: Mindfulness-based cancer recovery
OR: Odds ratios
RCTs: Randomized controlled trials
TCQ: Taichi/Qigong
WLC: Wait-list control
